# Redescriptions of the poorly known crane fly species *Tipula* (*Vestiplex*) *scandens* and *Tipula* (*Vestiplex*) *subscripta* from Tibet and Yunnan, China (Diptera, Tipulidae)

**DOI:** 10.3897/zookeys.917.38044

**Published:** 2020-03-09

**Authors:** Pavel Starkevich, Qiu-Lei Men, Duncan Sivell

**Affiliations:** 1 Nature Research Centre, Akademijos str. 2, LT–10222 Vilnius, Lithuania Nature Research Centre Vilnius Lithuania; 2 School of Life Sciences, Provincial Key Laboratory of the Biodiversity Study and Ecology Conservation in Southwest Anhui; Research Center of Aquatic Organism Conservation and Water Ecosystem Restoration in Anhui Province, Anqing Normal University, Anqing 246011, Anhui, China Anqing Normal University Anqing China; 3 Natural History Museum, Cromwell Road, London, SW7 5BD, UK Natural History Museum London United Kingdom

**Keywords:** taxonomy, biodiversity, systematics, hypopygium, ovipositor, *coquillettiana* species group, *
scripta* species group

## Abstract

Tipula (Vestiplex) scandens Edwards, 1928 and Tipula (Vestiplex) subscripta Edwards, 1928 were both briefly described based on single specimens and lacked illustration in the original literature. In the present paper, these two species are redescribed with new illustrations of additional morphological features based on type and non-type specimens.

## Introduction

Tipula (V.) scandens and T. (V.) subscripta were both described by Edwards (1928) based respectively on a single female specimen from Tibet and a single male from Yunnan, China. The original descriptions were very brief and lacked illustrations. Additional material was collected from Tibet and its adjacent areas by Frank Kingdon-Ward and Ronald J. H. Kaulback in 1933 during their expedition to South-eastern Tibet and by Kaulback in 1935–1936 (Alexander 1963). These specimens were subsequently identified and published by Alexander (1953, 1963), but no additional notes and illustrations were provided. The lack of detailed descriptions and illustrations has resulted in a limited understanding of these two species. In the present work, both sexes of T. (V.) scandens and T. (V.) subscripta are redescribed and illustrated. High quality photographs are also provided to facilitate identification.

## Materials and methods

Descriptive terminology generally follows that of Alexander and Byers (1981). The term gonocoxal fragment for the inner structure covered by tergite nine is adopted from Brodo (2017). Neumann (1958) designated the same structure as sclerites *sp1* and *sp2* and Dobrotworsky (1968) as the genital bridge.

The terminalia were removed and macerated in 10% NaOH for 5–10 minutes, observed in glycerin under an Olympus SZX10 stereomicroscope and preserved in microvials, filled with glycerol, on the same pin as the dry insect. Dry specimens were photographed with a Canon EOS 80D at the Natural History Museum, London. Digital photos were processed and layers were stacked using the program HeliconFocus (http://www.heliconsoft.com/heliconsoft-products/helicon-focus/).

For citing label data on specimens, a slashed line (/) separates each label. Square brackets ([]) are used to indicate additional information not on the original label.

Abbreviations for institutional collections used herein are: **BMNH** = Natural History Museum, London, United Kingdom; **USNM** = United States National Museum, Washington, D.C., USA.

## Taxonomy

### Tipula (Vestiplex)

Taxon classificationAnimaliaDipteraTipulidae

Bezzi, 1924

7E99BF75-4B6B-5A2E-BD24-7538BF1234DB

Tipula (Vestiplex) Bezzi, 1924: 230; Edwards, 1931: 79; Alexander, 1934: 396; 1935: 117; 1965: 355; Mannheims, 1953: 116; Savchenko, 1964: 132.

#### Type species.

*Tipula cisalpina* Riedel, 1913.

#### Notes.

*Vestiplex* was first proposed by Bezzi (1924) as a subgenus of *Tipula* for the type species *T. cisalpina* Riedel, 1913 recorded from the western Palaearctic (Italy, Switzerland). The subgenus T. (Vestiplex) is a phylogenetically young crane fly complex formed in the neo-paleogene (Savchenko 1960, 1964). No fossil species of T. (Vestiplex) are described so far and only Matthews and Telka (1997) mentioned ovipositors of possibly T. (Vestiplex) females from Cape Deceit Formation in Western Alaska (1.8 Ma old). The world fauna of the subgenus Tipula (Vestiplex) includes 173 species group taxa which are distributed throughout the Holarctic and Oriental Regions (Oosterbroek 2019). A total of 75 species group taxa are known from the Oriental Region with numerous accounts from China, India and Nepal (Oosterbroek 2019). The male genitalia are extremely polymorphic (Savchenko 1964), typically with the ninth tergite forming a shallowly concave and sclerotised saucer, other species have their ninth tergite completely divided longitudinally by a pale membrane (Alexander 1935, Alexander and Byers 1981). Females belonging to T. (Vestiplex) are characterized by a powerful, heavily sclerotized cercus, that is serrated along the outer margin (though smooth in several Asiatic species) and small to rudimentary hypovalva (Alexander 1935, 1965, Alexander and Byers 1981).

### Tipula (Vestiplex) scandens

Taxon classificationAnimaliaDipteraTipulidae

Edwards, 1928

4493EECC-7817-52AC-A772-94028D7E58D3

Figures 1 , 2–12 , 13 , 14–16

Tipula scandens Edwards, 1928: 691; Edwards, 1931: 80; Alexander, 1935: 119; Wu, 1940: 15; Alexander, 1953: 343; Savchenko, 1964: 228; Oosterbroek and Theowald 1992: 158.

#### Diagnosis (male).

Tipula (V.) scandens can be recognized by following combination of characters: body blackish antenna long, reaching the first abdominal segment if bent backwards, the first flagellomere cylindrical, the remaining flagellomeres strongly dilated at both ends, hypopygium with tergite nine in the shape of a narrow, transverse, sclerotised saucer-shaped plate, gonocoxite unarmed.

#### Redescription.

**Male.** Body length: 10.9–15.6 mm. Wing: 12.6–15.2 mm.

**Figure 1.**
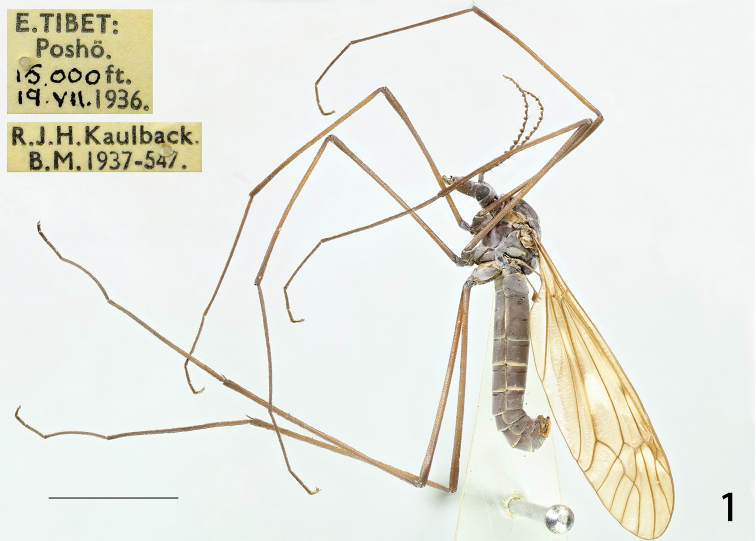
Male habitus of T. (V.) scandens, lateral view.

Head. Blackish in general. Rostrum blackish, thinly dusted with grey, nasus indistinct (Fig. 1). Vertex and occiput blackish, grey pruinose, with a narrowed black line medially. Antenna 13-segmented, if bent backwards almost reaching the posterior margin of the first abdominal segment; scape blackish, narrowed basally and broadened apically, pedicel blackish, very short; flagellum blackish with first flagellomere cylindrical, slightly curved, remaining flagellomeres strongly dilated at both ends, gradually shortening in length, the last flagellomere very small, basal enlargement of flagellomeres with five black verticils, verticils slightly shorter than the length of the corresponding flagellomeres (Fig. 1). Palpus entirely blackish.

Thorax. Pronotum blackish, white pruinose medially. Prescutum blackish, grey pruinose, with three blackish stripes, the laterals oblong, bordered by black line, the median one expanded apically and narrowed at base, separated by a black median line. Scutum blackish, grey pruinose, with two black spots. Scutellum blackish, grey pruinose. Mediotergite blackish. Pleura densely dusted with grey, only suffused with brown at the border of dorsum and pleura (Fig. 1). Leg with coxa blackish, thinly dusted with grey; trochanter black, femur and tibia dark brown with darker tips, two basal tarsal segments dark brown, remaining brownish black (Fig. 1). Wing yellowish-brown, cells c and sc not darker than ground colour, wing cells marbled with light brown spots scattered at base of Rs and around the stigma; with brown clouds in the arcular area, and distal and middle areas of bm, the latter extended along Cu; apical cells also suffused with light brown. Rs relatively short, subequal in length to R_3_, R_1+2_ entire, discal cell broad, elongated, petiole of cell m_1_ indistinct (Fig. 1). Halter with stem dark brown, knob blackish (Fig. 1).

**Figures 2–12.**
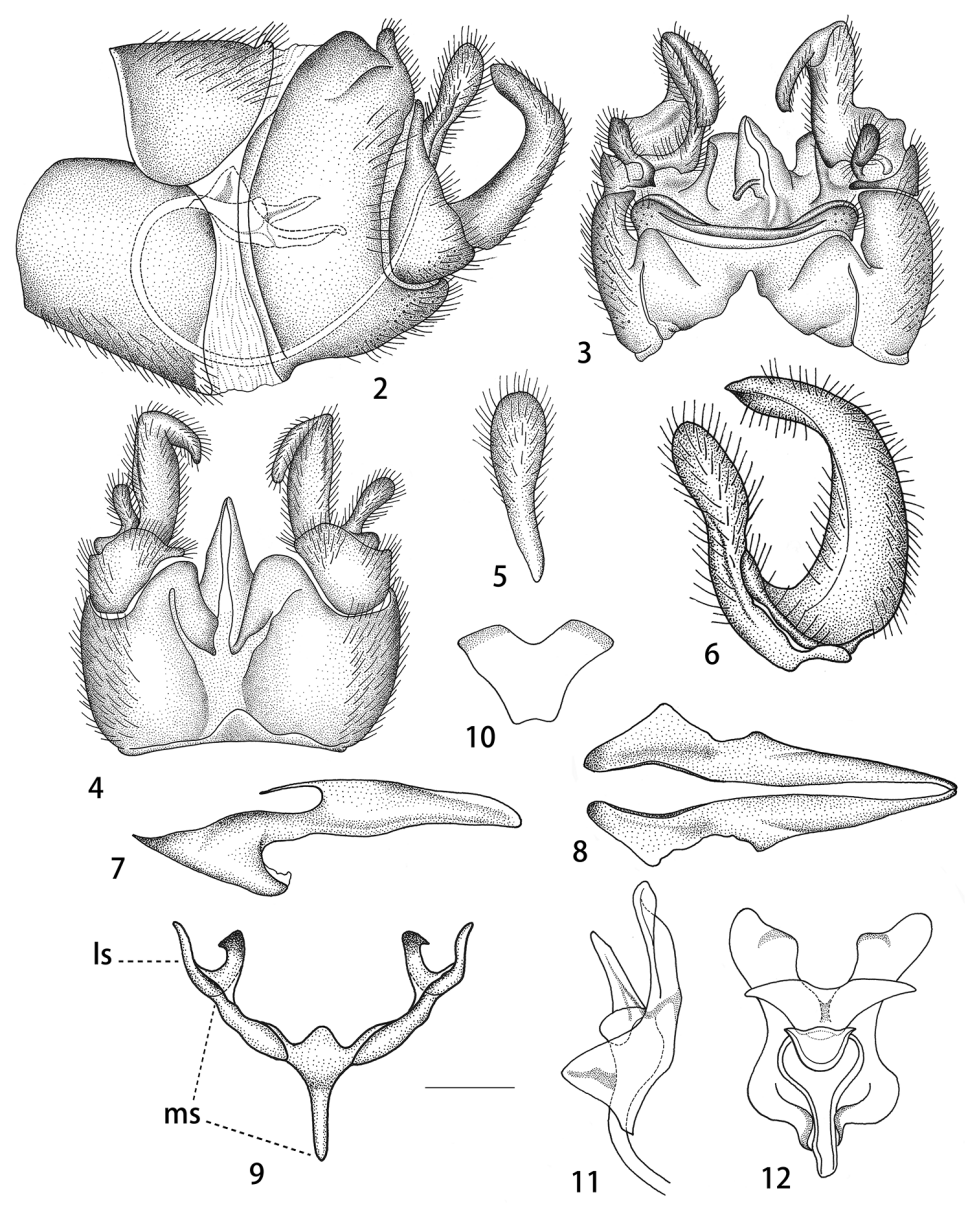
Male hypopygium of T. (V.) scandens. **2** Hypopygium, lateral view **3** hypopygium, dorsal view **4** hypopygium, ventral view **5** outer gonostylus, lateral view **6** outer gonostylus and inner gonostylus, lateral view **7** adminiculum, lateral view **8** adminiculum, ventral view **9** gonocoxal fragment, dorsal view **10** compressor apodeme of semen pump **11** semen pump, lateral view **12** semen pump. Abbreviations: ls, lateral sclerite of gonocoxal fragment; ms, medial sclerite of gonocoxal fragment. Scale bars: 0.4 mm (**2–4**), 0.25 mm (**5–12**).

Abdomen. Abdomen including hypopygium blackish, segments two to four narrowly suffused with brown on posterior margin (Fig. 1). *Hypopygium.* Tergite nine separated from sternite nine by a distinct narrowed notch (Figs 2, 3). Tergite nine in the shape of a narrow, transverse, sclerotised saucer-shaped plate (Fig. 3). The main body of tergal saucer brownish. Posterior margin of tergal saucer is broadly emarginated, lateral lobes rounded, densely covered with setae, anterior portion with raised border appearing as elevated transverse long and very narrow plate almost reaching lateral lobes. Sternite nine broad, distinctly protruding at hind margin, deeply divided by a V-shaped notch (Fig. 4). Gonocoxite triangular, unarmed (Fig. 2). Outer gonostylus narrowed at base, gradually broadened to the round apex (Fig. 5). Inner gonostylus a claw-shaped curved plate (Figs 2–4, 6). Beak extended into blackened obtuse rostrum. Posterior parts of medial sclerite lightly flattened. Lateral sclerites U-shaped. Adminiculum triangular tube-shaped, surpassing the end of gonocoxite, acute apically, with a triangular process at base in lateral view (Figs 7, 8). Gonocoxal fragment with medial sclerite V-shaped, at base with narrow apodeme, posteriorly rounded (Fig. 9). Semen pump very small, situated between eighth and ninth segments. Compressor apodeme fan-shaped with a V-shaped notch medially, the apical margin narrowly suffused with black (Fig. 10). Posterior immovable apodeme distinctly longer than compressor apodeme, very enlarged in dorsal view (Figs 11, 12). Anterior immovable apodeme short, gradually narrowed to apex, with a black stripe medially in lateral view (Figs 11, 12). Aedeagus tubular, almost three times as long as semen pump, thickened at base and gradually narrowed to end, acute apically (Fig. 2).

**Figure 13.**
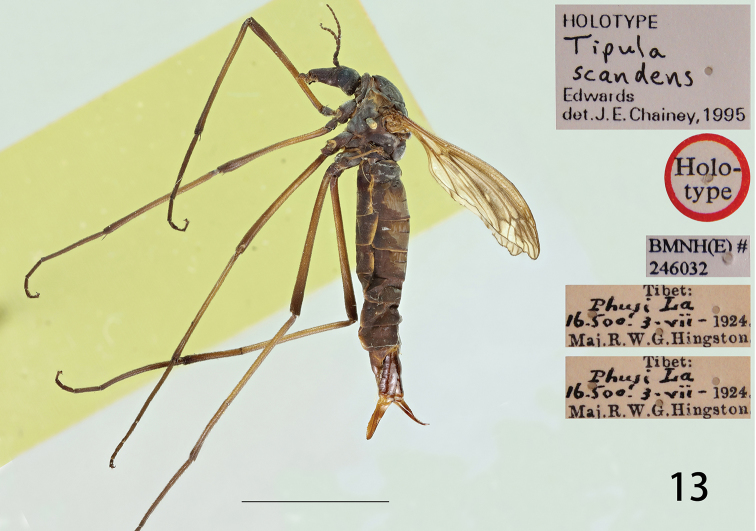
Female habitus of T. (V.) scandens (holotype), lateral view.

**Female.** Body length: 15.6–18.4 mm. Wing: 8.0–8.1 mm. Body colour same as that of male except as follows. Antenna relatively short, if bent backward reaching before base of the wing. Scape expanded apically and narrowed basally, pedicel short, flagellum with flagellomeres cylindrical, gradually shortening in length distinctly shorter than the length of the corresponding flagellomeres (Fig. 13). Legs stout, femora distinctly dilated towards the apex (Fig. 13). Wing reduced, never more than half the body length (Fig. 13). Abdomen dark brown, tergites two to four with a pale median area, sternites two to four suffused with light brown on the lateral and posterior borders (Fig. 13).

**Figures 14–16.**
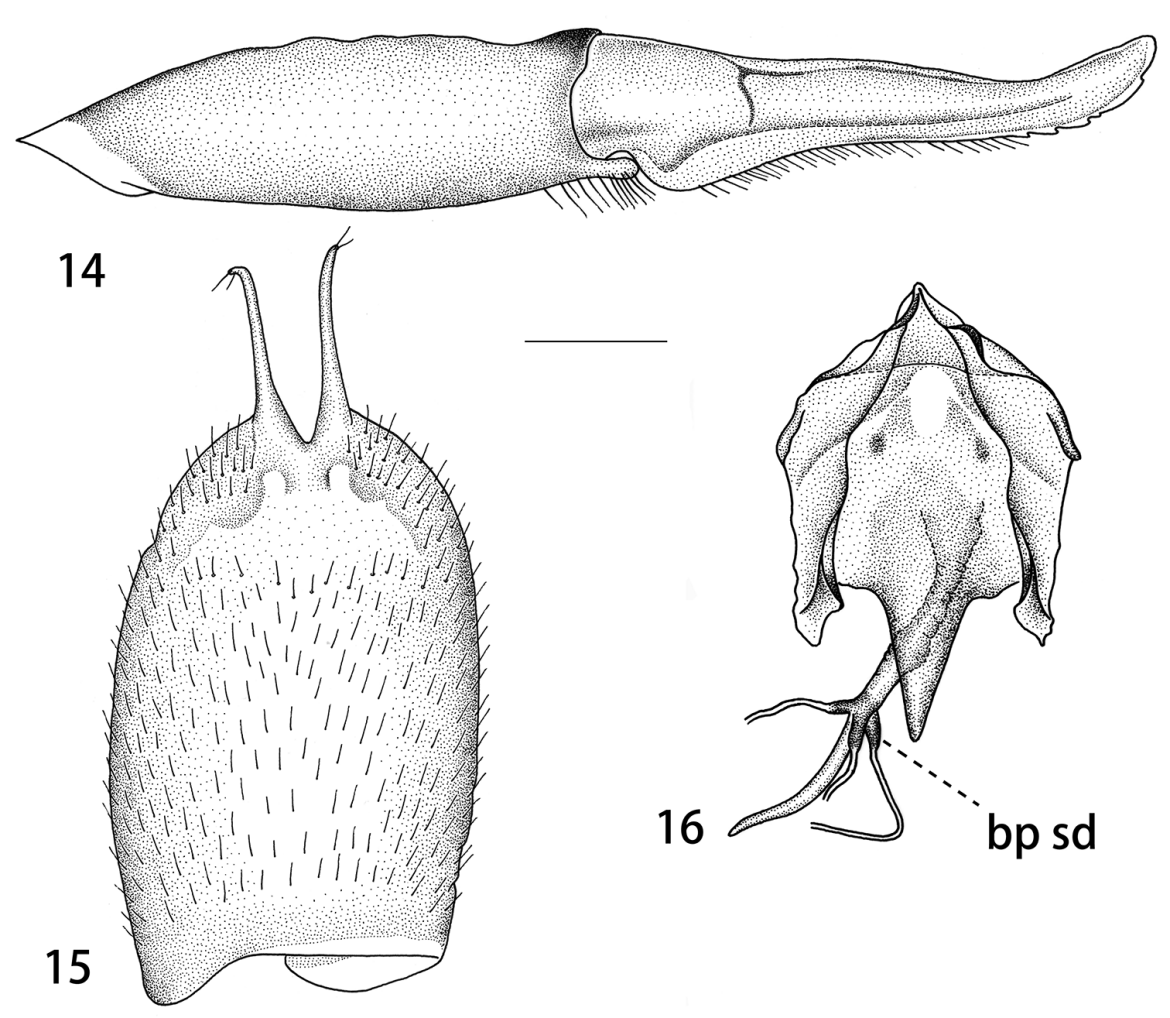
Female ovipositor of T. (V.) scandens. **14** Tergite ten and cercus, lateral view **15** sternite eight with hypovalvae, ventral view **16** sternite nine, furca, and part of internal reproductive system, dorsal view. Abbreviation: bp sd, basal part of spermathecal duct. Scale bars: 0.5 mm (**14, 15**), 0.4 mm (**16**).

Ovipositor. Tergite nine dark brown, tergite ten shining dark brown. Cercus brown, with tip narrowed and slightly up-turned, outer margin with rough serration (Fig. 14). Hypovalva reduced, filamentous, slender, terminating in two setae (Fig. 15). Median incision between hypovalvae slightly deeper than posterior margin of sternite eight. Lateral incision absent. Sternite nine posteriorly triangular, anterior parts flattened (Fig. 16). Furca posteriorly flattened, posteriorly triangular in shape (Fig. 16). Bursa copulatrix with spermathecal duct sclerotised at base, in the shape of short, swollen, brown process (Fig. 16). Wall of bursa copulatrix sclerotised at connection site with spermathecal duct. Sclerotisation of all three spermathecal ducts connected and forming a complete dark brown ring. Anterior part of bursa copulatrix roughly straight.

#### Type material examined.

Holotype, female, **China**: “Type” / “Tibet: Phusi La 16, 500’, 3- vii-1924. Maj. R. W. G. Hingston” / “*Tipula scandens* Edw F.W.Edwards. det. 1928” / “HOLOTYPE” / “HOLOTYPE *Tipula scandens* Edwards det. J. E. Chainey, 1995” / “BMNH(E)#246032”.

#### Additional material examined.

Tipula (Vestiplex) scandens Edwards, 1928: **China**: 1 female, E. Tibet, Poshö, 16,000 ft., 20. VII. 1936, R. J. H. Kaulback, B. M. 1937-547, *Tipula scandens* Edw. Det. C. P. Alexander 1950 (USNM); 2 males, East Tibet, DüChu Valley, 14,000 ft, 10–15.vii.1936, R. J. H. Kaulback, B. M. 1937-547 (BMNH); 1 male, East Tibet, DüChu Valley,13,000 ft., 10–15. vii. 1936, R. J. H. Kaulback. B. M. 1937-547 (BMNH); 3 males, East Tibet, Poshö, Dzongra, 14.500 ft, 4. vii. 1936, R. J. H. Kaulback, B. M. 1937-547, *Tipula scandens* Edw. Det. C. P. Alexander 1950 (BMNH); 1 male, East Tibet, Poshö Dzongra, 15,000 ft., 4. vii. 1936, R. J. H. Kaulback, B. M. 1937-547, *Tipula scandens* Edw. Det. C. P. Alexander 1950 (BMNH).

#### Distribution.

China (Tibet).

#### Remarks.

T. (V.) scandens belongs to the *coquillettiana* species group (Savchenko 1960). According to Savchenko (1960) males are characterized by a tergite nine that is in the shape of a narrow, transverse sclerotised plate, its posterior margin broadly emarginated, with the lateral angles usually produced into an obtuse ledge. Anterior border of tergite nine elevated into lightly curved long edge reaching the lateral angles of tergite nine.

### Tipula (Vestiplex) subscripta

Taxon classificationAnimaliaDipteraTipulidae

Edwards, 1928

E1F171DF-1EF3-53CE-A91A-AD42CC7034C0

Figures 17 , 18–30 , 31 , 32–33

Tipula subscripta Edwards, 1928: 689; Edwards, 1931: 80; Alexander, 1935: 119; Wu, 1940: 15; Alexander, 1963: 327; Savchenko, 1964: 164; Alexander and Alexander 1973: 65; Oosterbroek and Theowald 1992: 159.

#### Diagnosis (male).

Tipula (V.) subscripta can be recognized by following combination of characters: body yellowish-brown, antenna bicolored except three yellow basal segments, if bent backwards reaching the base of the wing, tergite nine forming a pale-yellow saucer-shaped plate anteriorly having raised border, gonocoxite armed with a strong spine.

#### Description.

**Male.** Body length: 14.4–14.7 mm. Wing: 18.2–19.1 mm.

**Figure 17.**
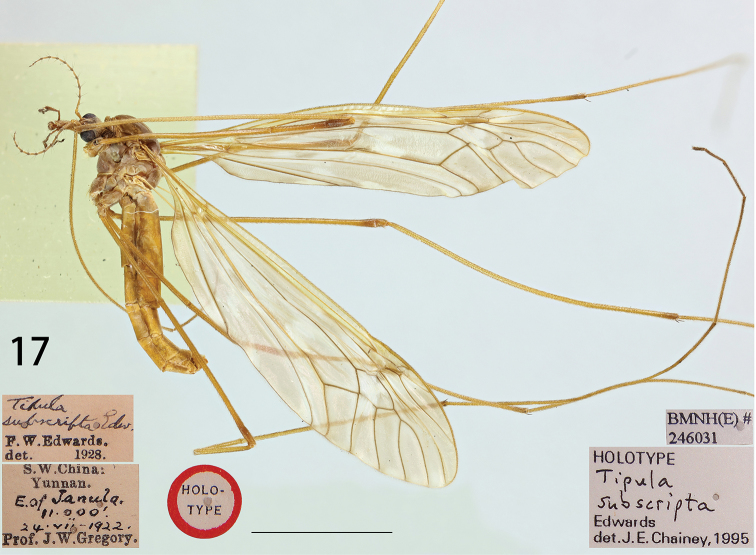
Male habitus of T. (V.) subscripta (holotype), lateral view.

Head. Yellow in colouration generally (Fig. 17). Rostrum yellow, nasus distinct and yellow. Vertex and occiput greyish, medially with brown line which is very narrow at the occiput becoming broad between the eyes. Antenna 13-segmented, if bent backwards just reaching the base of the wing; scape yellow, expanded apically and narrowed basally, pedicel yellow and short, flagellum with first flagellomere entirely yellow, slightly narrowed at apex, the remaining flagellomeres bicoloured, brown basally and yellow apically, each flagellomere gradually shortening in length, basal enlargements with abundant black verticils, almost subequal in length to their corresponding flagellomeres (Fig. 17). Palpus dark brown.

Thorax. Pronotum yellow, black in middle and whitish pruinose laterally. Prescutum brownish with four grey stripes, the lateral stripes narrow with broad brown border, the median two stripes also suffused with brown borders. Space between median and lateral stripes whitish pruinose. Scutum yellowish brown, thinly whitish pruinose, with two grey spots, the posterior one distinctly bigger than anterior one. Scutellum and mediotergite yellowish brown, whitish pruinose, both with a brown median line. Pleura brownish, darker on dorsal side of anepisternum. Leg slender, coxa same as pleuron in colouration, trochanter yellow, femur and tibia yellow with slightly dark tips, tarsus yellowish brown (Fig. 17). Wing relatively transparent, cells c and sc not darker than the ground colour, wing cells marbled with light brown spots scattered by base and apex of Rs and around the stigma, two brown clouds distributed along Cu. Rs relatively short, subequal in length to R_3_, R_1+2_ entire, discal cell broad, at least four times as long as the petiole of cell m_1_ (Fig. 17). Halter with stem yellow, knob yellowish brown.

**Figures 18–30.**
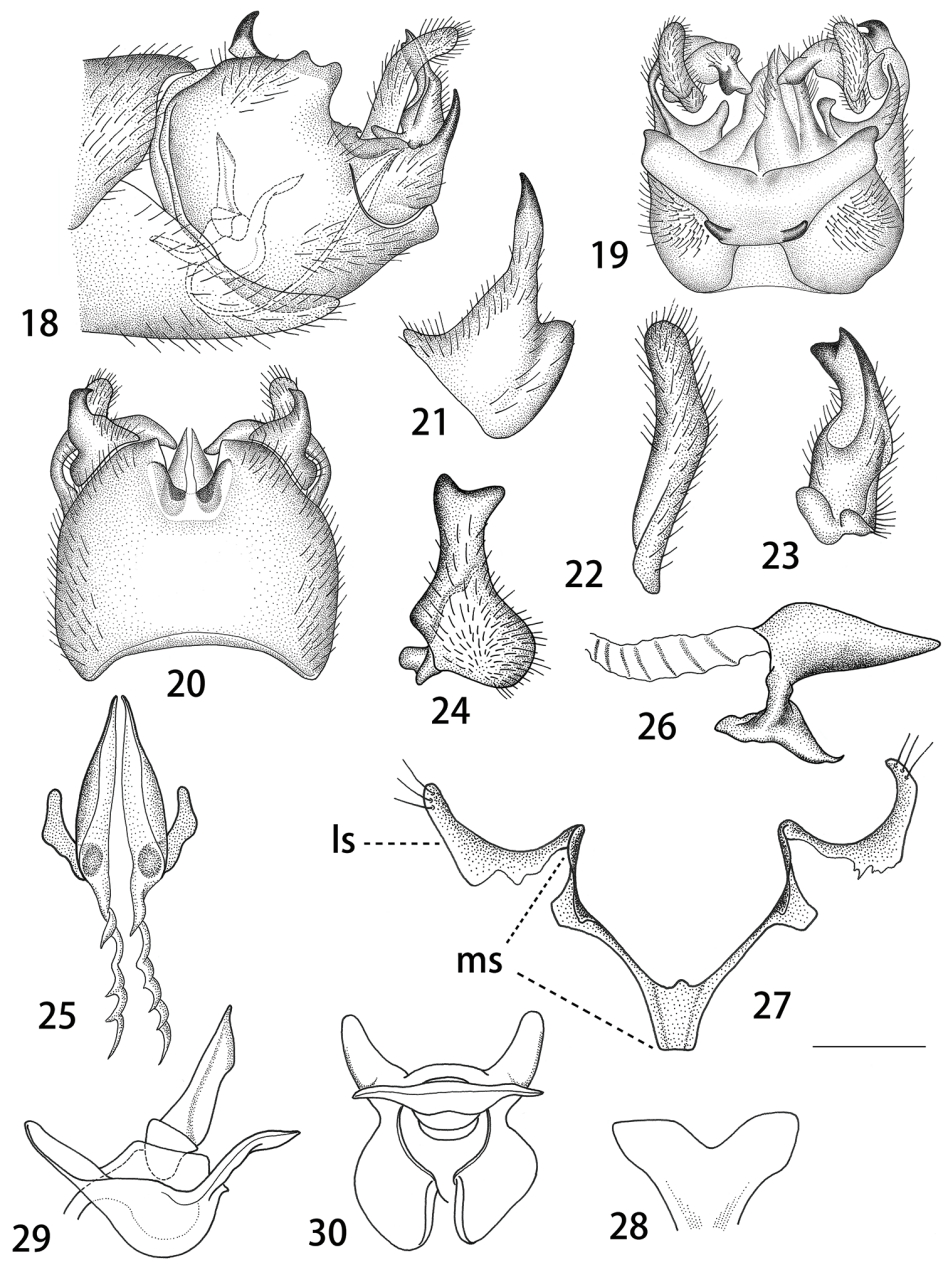
Male hypopygium of T. (V.) subscripta. **18** Hypopygium, lateral view **19** hypopygium, dorsal view **20** hypopygium, ventral view **21** gonocoxite, lateral view **22** outer gonostylus, lateral view **23** inner gonostylus, outer lateral view **24** inner gonostylus, inner lateral view **25** adminiculum, ventral view **26** adminiculum, lateral view **27** gonocoxal fragment, dorsal view **28** compressor apodeme of semen pump **29** semen pump, lateral view **30** semen pump. Abbreviations: ls, lateral sclerite of gonocoxal fragment; ms, medial sclerite of gonocoxal fragment. Scale bars: 0.4 mm (**18–20**), 0.25 mm (**21–30**).

Abdomen. First abdominal segment yellowish, remaining segments including hypopygium yellowish-brown (Fig. 14). *Hypopygium.* Tergite nine mostly fused with sternite nine (Figs 18, 19). Tergite nine distally forming a pale-yellow saucer-shaped plate (Fig. 19). The main body of tergal saucer is pale-yellow. Posterior margin of tergal saucer is broadly emarginated, posterior lobes pale laterally with incision. Anterior portion brown with raised border appearing as an elevated transverse quadrate plate. The quadrate plate medially pale, lateral angles produced into black tooth. Sternite nine broad and rounded in general, with a U-shaped notch at hind margin (Fig. 20). Gonocoxite armed with a strong spine, apex acute and blackened (Figs 18, 21). Outer gonostylus lightly curved, finger-shaped (Fig. 22). Inner gonostylus in the shape of slightly curved plate, bifid at apex, dorsally with a black obtuse tooth, beak extended into blackened obtuse rostrum (Figs 23, 24). Posterior parts of medial sclerite distally flattened. Lateral sclerites U-shaped. Adminiculum in the shape of short, triangular tube (Figs 25, 26). Gonocoxal fragment with medial sclerite slender, V-shaped, with flattened and short apodeme at base (Fig. 27). Semen pump situated between eighth and ninth segments. Compressor apodeme fan-shaped, medially with a V-shaped notch (Fig. 28). Posterior immovable apodeme subequal in length to compressor apodeme, rounded apically in dorsal view (Figs 29, 30). Anterior immovable apodeme short, gradually narrowed to apex in lateral view, an expanded lobe in dorsal view (Figs 29, 30). Aedeagus tubular, almost two times as long as semen pump, thickened at base and gradually narrowed to end, acute apically (Fig. 18).

**Figure 31.**
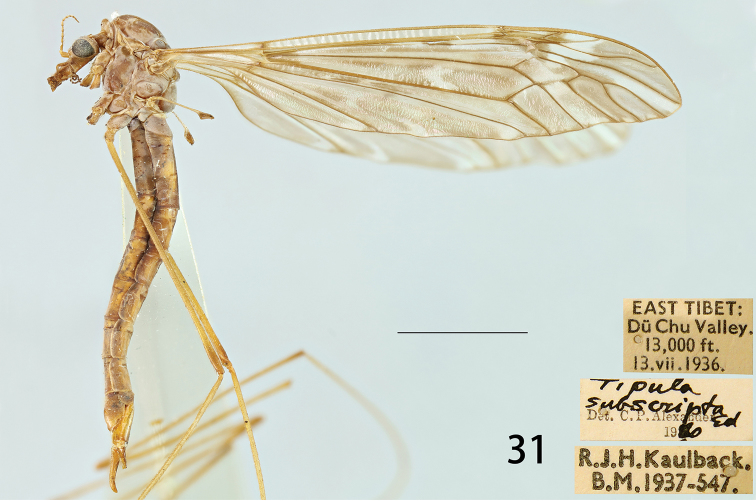
Female habitus of T. (V.) subscripta, lateral view.

**Female.** Body length: 21.9–24.5 mm. Wing: 20.7–23.1 mm. Body colouration same as that of male except as follows. Antenna, if bent backward reaching pronotum. Scape and pedicel yellow, flagellar segments darkened at base. Each flagellomere slightly narrowed at apex, gradually shorter in length, base with five black verticils that are slightly longer than the length of its corresponding flagellomere (Fig. 31). Leg with femur and tibia darker at apex than that of male (Fig. 31). Wing darker in colouration than that of male, with scattered light brown spots at base of Rs and around the stigma, with light brown clouds distributed as follows: arcular area, median area of br cell, median and apical areas of bm cell, a_1_ and a_2_ cells, apical half of r_1+2_ and r_3_ cells, median area of r_4+5_ cell (Fig. 31). Abdomen yellowish brown, with brown dorsomedial and lateral stripes.

**Figures 32–34.**
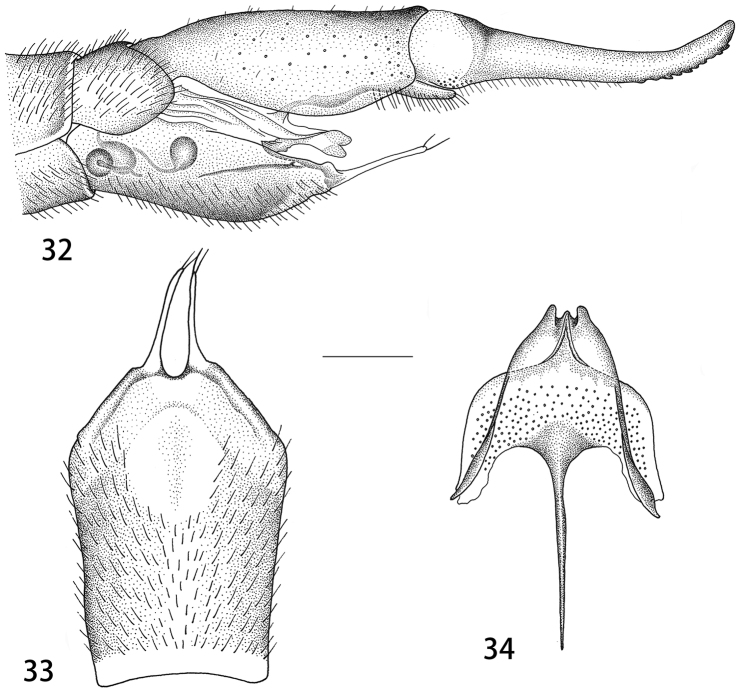
Female ovipositor of T. (V.) subscripta. **32** Ovipositor, lateral view **33** sternite eight with hypovalvae, ventral view **34** sternite nine and furca, dorsal view. Scale bars: 0.6 mm (**32**), 0.5 mm (**33**), 0.25 mm (**34**).

Ovipositor. Tergite nine brown, tergite ten shining brown. Cercus brown, slender, with tip narrowed and up-turned, outer margin with rough serration (Fig. 32). Hypovalva reduced, filamentous, slender, terminating in two setae (Fig. 33). Median incision between hypovalvae slightly deeper than posterior margin of sternite eight. Lateral incision absent. Sternite nine posteriorly with two short extensions on either side beneath the apex, anterior parts narrow (Fig. 34). Furca posteriorly pale, anteriorly brown, long and narrow.

#### Type material examined.

Holotype, male, **China**: “Type” / “S. W. China: Yunnan. E. of Janula. 11,000’, 24. vii-1922. Prof. J. W. Gregory” / “*Tipula subscripta* Edw. F. W. Edwards. det. 1928” / “HOLOTYPE” / “HOLOTYPE *Tipula subscripta* Edwards det. J. E. Chainey, 1995” / “BMNH (E) #246031”.

#### Additional material examined.

Tipula (Vestiplex) subscripta Edwards, 1928: **China**: 1 female, Southeast Tibet, Nagong, Shiuden Gompä, 12,500 ft., 25. viii. 1933, F. Kingdon Ward, B. 14,000 ft., 24. VII. 1936, R. J. H. Kaulback, B. M. 1937-547, *Tipula subscripta* Ed. Det. C. P. Alexander 19[??] (BMNH). Abdomen and leg with antena on two separate slides (USNM); 1 male, East Tibet, Poshö, 13,000 ft., 29. VII. 1936, R. J. H. Kaulback, B. M. 1937-547, *Tipula subscripta* Ed. Det. C. P. Alexander 1936 (BMNH); 1 female, Southeast Tibet, Shugden Gompä 13,000 ft., 18. VIII. 1935, R. J. H. Kaulback, B. M.1937-547, *Tipula subscripta* Ed. Det. C. P. Alexander 1960 (BMNH); 2 females, Southeast Tibet, Nagong, Shiuden Gompä, 12,000 ft., 25. viii. 1933, F. Kingdon Ward, B. M. 1934-155, *Tipula subscripta* Ed. Det. C. P. Alexander 1960 (BMNH); 1 female, East Tibet, DüChu Valley, 13,000 ft., 13. vii. 1936, R. J. H. Kaulback, B. M.1937-547, *Tipula subscripta* Ed. Det. C. P. Alexander 1960 (BMNH).

#### Distribution.

China (Yunnan, Tibet).

#### Remarks.

T. (V.) subscripta belongs to the *scripta* species group. The *scripta* species group was proposed by Mannheims (1953), discussed by Savchenko (1960, 1964), and the range of species were revised by Starkevich and Podenas (2011, 2015). Males of the *scripta* group are characterized by the following features: gonocoxite horn-shaped, tergite nine forming a pale-yellow saucer-shaped plate with posterior margin emarginated and anterior portion appearing as an elevated transverse quadrate plate.

## Supplementary Material

XML Treatment for Tipula (Vestiplex)

XML Treatment for Tipula (Vestiplex) scandens

XML Treatment for Tipula (Vestiplex) subscripta
